# S5Utis: Structured State-Space Sequence SegNeXt UNet-like Tongue Image Segmentation in Traditional Chinese Medicine

**DOI:** 10.3390/s24134046

**Published:** 2024-06-21

**Authors:** Donglei Song, Hongda Zhang, Lida Shi, Hao Xu, Ying Xu

**Affiliations:** 1 College of Computer Science and Technology, Jilin University, Changchun 130012, China; dongleisong93@foxmail.com (D.S.);; 2 School of Artificial Intelligence, Jilin University, Changchun 130012, China

**Keywords:** tongue image segmentation, traditional Chinese medicine, tongue diagnosis, semantic segmentation

## Abstract

Intelligent Traditional Chinese Medicine can provide people with a convenient way to participate in daily health care. The ease of acceptance of Traditional Chinese Medicine is also a major advantage in promoting health management. In Traditional Chinese Medicine, tongue imaging is an important step in the examination process. The segmentation and processing of the tongue image directly affects the results of intelligent Traditional Chinese Medicine diagnosis. As intelligent Traditional Chinese Medicine continues to develop, remote diagnosis and patient participation will play important roles. Smartphone sensor cameras can provide irreplaceable data collection capabilities in enhancing interaction in smart Traditional Chinese Medicine. However, these factors lead to differences in the size and quality of the captured images due to factors such as differences in shooting equipment, professionalism of the photographer, and the subject’s cooperation. Most current tongue image segmentation algorithms are based on data collected by professional tongue diagnosis instruments in standard environments, and are not able to demonstrate the tongue image segmentation effect in complex environments. Therefore, we propose a segmentation algorithm for tongue images collected in complex multi-device and multi-user environments. We use convolutional attention and extend state space models to the 2D environment in the encoder. Then, cross-layer connection fusion is used in the decoder part to fuse shallow texture and deep semantic features. Through segmentation experiments on tongue image datasets collected by patients and doctors in real-world settings, our algorithm significantly improves segmentation performance and accuracy.

## 1. Introduction

With the advancement of science and technology and the improvement of living standards, people are more clearly feeling the pressure from high-intensity work and study and are paying more attention to their health status. People’s management of their own health has also gradually transitioned from seeking medical treatment after symptoms appear, to preventive health care focusing on physical examinations [1]. In terms of maintaining good health through scientific exercise, there are health-care behaviors with Chinese characteristics, such as square dancing and other health-care exercise activities. People have a more urgent need to actively understand their own health status, actively obtain health knowledge, and obtain timely health feedback. The development of science and technology, especially the popularization of smart devices and the development of artificial intelligence technology, has provided feasibility for this series of needs [1,2]. Traditional health examinations require participants to go to designated medical facilities and undergo item-by-item examinations, including some invasive ones. This often takes people a day to complete, and the physical examination report is received some time after the examination. Although physical examination is a very effective way to manage health, the shortage of medical resources and of certain examinations that have an impact on the human body during the physical examination are among the reasons that physical examination is only recommended as a regular health management method. People’s need to know their health status at any time is still unsatisfied. Smartphones are devices with rich sensors that can be used anytime and anywhere, and are an excellent way to collect physiological information [3]. We hope to help people better participate in medical and health activities by using the sensors in smartphones. In the research on using smartphone sensors to assist health checks and healthy living, mobile phone cameras are one of the most commonly used sensor devices. Mobile phone cameras are widely used in medical and health care activities such as skin health and eye health due to their high imaging quality, ease of use, and easy access [3].

Traditional Chinese Medicine (TCM) is an important and popular part of the Chinese medical system, with a profound theoretical foundation and practical application [4]. It has now become an essential part of the World Health Organization’s alternative medicine program [5,6]. TCM is more easily accepted by people because of its closeness to life medical philosophy and non-invasive treatment methods. The “2030 Shanghai Declaration on Health Promotion in Sustainable Development” proposed to “Consider the growing importance and value of traditional medicine, which could contribute to improved health outcomes, including those in the sustainable development goals (SDGs)” [7]. TCM has a long history of application in China, with wide acceptability and long-term research in health management. We hope to assist in health management by providing intelligent traditional Chinese medicine services anytime and anywhere by using smart TCM to improve people’s health awareness, encourage people to actively participate in daily health management actions, recognize the importance of health, gain motivation to improve health, and help the sustainable development of health management. In TCM, diagnosis is usually divided into two stages: the first stage is ‘syndrome differentiation’, and the second is ‘treatment’. In TCM, the practitioner needs to first observe and summarize the patient’s physical condition and symptoms before they can recommend treatment methods. Treatment methods such as Chinese herbal medicine and acupuncture are usually used. Therefore, it is crucial to obtain a thorough and detailed picture of the patient’s status before treatment begins. The process of ‘inspection, auscultation–olfaction, inquiry, and palpation’ is routinely used in TCM diagnostics. Through the above four steps, patient information is collected; after comprehensive analysis, a suitable judgment of the patient’s constitution and disease is then completed [8]. Tongue diagnosis, as the first step in the inspection stage, has very important practical significance [9,10]. Based on the theory of TCM, the observation and analysis of tongue characteristics (e.g., the shape, color, tongue coating status, etc.) can help doctors to obtain intuitive feedback on the patient’s physical condition to a certain extent. Considering the easy accessibility of smartphones and the simplicity and ease of using mobile phone cameras to collect physiological information, using smartphone cameras for intelligent tongue diagnosis in traditional Chinese medicine is a very valuable research topic. During the COVID-19 pandemic, tongue diagnosis became an effective method to assist diagnosis [11,12,13,14]. When it is inconvenient for people to go to the hospital for medical examinations, remote tongue diagnosis using smartphones can become a very effective supplementary medical method. By using the mobile phone camera to take a picture of the tongue, people can receive diagnosis results and corresponding medical advice.

As people’s demand for smart medical care is increasing and the development of intelligent medicine continues to accelerate, a great deal of research has been conducted on intelligence and objectification in traditional medicine. In intelligent TCM diagnosis, automated tongue diagnosis is more conducive to applications in daily healthcare than other diagnostic methods due to its wide availability, non-invasiveness, good application history, and strong correlation with the overall condition of the body. As an important facet of intelligent TCM, intelligent tongue diagnosis has attracted the attention of many researchers [15]. Generally, the process of intelligent tongue diagnosis consists of four steps: tongue image acquisition, tongue image segmentation, tongue image feature extraction, and tongue image classification. This process is followed to complete tasks such as the identification and classification of the tongue shape, tongue color, coating color, tongue texture, and any corresponding ‘syndrome’ [16]. Tongue image segmentation is a crucial step, as the effect of segmentation will directly affect subsequent tongue image feature analysis and classification, such as in the analysis of tongue shape and tongue texture. Tongue image segmentation can be defined as extracting the tongue part from the facial image containing the tongue and removing other noise. Unlike the black-and-white images of typical medical image segmentation modalities such as X-ray or CT, tongue images are rich in color. Tongues come in a variety of colors and shapes, and are similar in color to surrounding features such as the neck, face, and lips, which increases the challenge of tongue segmentation.

Many algorithms for tongue image segmentation are mostly used on tongue images taken in standard environments collected by professional tongue diagnostic instruments [17,18]. Due to the use of standard lighting, professional operation, and high imaging quality, these tongue images are usually of high quality and make it easier to complete the task of tongue segmentation. In the process of promoting the comprehensive application of intelligent TCM, using portable devices such as mobile phones and other smart devices to collect tongue image data more conveniently is an important means of building TCM data sets [19]. A number of researchers have discussed the tongue image data obtained in the open environment and corrected the color change problem by optimizing the network structure. Li et al. proposed a lightweight segmentation algorithm that can be applied to tongue images taken by mobile phones. In the encoder part, dilated separable convolution, cascaded convolution, and ASPP are combined for feature extraction [20]. In order to overcome the interference of illumination on tongue shape color and affect the segmentation effect, Gao et al. [21] proposed a level set model which was used to train the color tongue image through a convolutional neural network to obtain an edge probability map, then combined with the symmetry of the tongue image and edge constraints based on the level set model to achieve better segmentation results when there large differences in color temperature and color difference are present between tongue images. However, as the person operating the mobile phone to collect tongue images may be a patient or a doctor conducting remote diagnosis for treatment or review, the collected tongue image data often contain a large amount of redundant content and noise, such as complete facial features, complex backgrounds, etc. Such pictures are difficult to use and analyze directly; thus, tongue image segmentation is also required in order to extract the tongue from the surrounding image. Due to factors such as differences in shooting equipment, the professionalism of the photographers, and the cooperation of the subjects, the size and quality of the captured images may vary. Large differences in shooting angles, insufficient tongue exposure, tongue twisting, a small proportion of the tongue in the image, and insufficient lighting can make it more difficult to distinguish the tongue from other noisy areas, increasing the challenge of tongue image segmentation. The CNN-based semantic segmentation method can automatically process large amounts of image data, significantly improving processing efficiency. The network is robust and performs well even in complex image environments. Traditional methods typically handle tongue images taken with professional equipment in controlled environments and are designed for high-quality datasets [22,23,24,25]. While they have shown positive results with good image quality, traditional non-CNN methods are highly sensitive to image quality and noise, and cannot adapt well to our scenario. In our case, the image quality is uneven and achieving high-quality processing in a short time with non-CNN models is challenging. CNN-based deep learning methods offer advantages that are more suitable for our scenario. Therefore, based on a comprehensive analysis of the existing research, we have decided to employ deep learning methods to address our problem. We compare the effectiveness of our proposed method with other deep learning approaches.

In response to the above tongue image segmentation problem, we propose a segmentation algorithm called S5Utis (Structured State-Space Sequence SegNeXt UNet-like Tongue Image Segmentation) for tongue images collected in complex multi-device and multi-user environments. Specifically, our model uses SegNeXt as the backbone and a convolutional attention mechanism [26] to perform multi-scale feature fusion of tongue images. The Structured State-Space Sequence Model (S4) [27] network is extended to a 2D environment to improve the convolutional attention block. Then, shallow texture features and deep semantic features are integrated through cross-layer connections and layer-by-layer upsampling is used to improve the resolution of decoding features to ensure that the tongue image segmentation details are not lost. In order to verify the effectiveness of the proposed method, we compare it with other centralized classical methods on the collected open environment datasets. Experimental results show that our method performs well in improving segmentation performance. Our work provides a positive approach to using mobile phones for intelligent TCM tongue diagnosis, allowing the camera sensors of smartphones to be better applied in medical and health practice.

The main contributions of this article are as follows:

- A tongue image segmentation model based on the SegNeXt network is proposed, with the S4 network as a more efficient and lightweight network backbone. It uses improved S4-2D convolutional self-attention for multi-scale feature fusion.
- Cross-layer connections and residual connections are used in the decoder to achieve layer-by-layer upsampling and improve segmentation accuracy.
- Better segmentation accuracy is achieved for tongue images taken by non-laboratory personnel and using non-professional equipment, which is an advantage over other currently popular semantic segmentation networks.

## 2. Related Work

### 2.1. Traditional Image Segmentation

Traditional tongue image segmentation methods usually rely on image processing techniques such as region segmentation, threshold-based segmentation, edge detection-based segmentation, or a combination of these methods for tongue image segmentation. Ning et al. [28] proposed a region merging-based automatic tongue segmentation method. They segmented the diffused tongue image, then used maximal-similarity-based region merging to extract the area of the tongue body. Zhang et al. [29] used a polar edge detector to extract the edge of the tongue body, introduced an edge filtering scheme to binarize object edges to eliminate noise caused by tongue color and other features, and finally used SNAKE to complete tongue image segmentation. Pang et al. [30] used the energy function-based bi-elliptical deformable template (BEDT) to capture the overall shape characteristics and roughly describe the shape of the tongue; then, after minimizing the energy function, they used the SNAKE algorithm to obtain the initial contour curve. Guo et al. [31] designed a method based on Otsu and two-stage k-means clustering for initial contour extraction by converting tongue images into the HSV color space and realized automatic contour recognition using the SNAKE algorithm. Wu et al. [32] proposed a segmentation method that combines region-based and edge-based methods. This method extracts ROIs (regions of interest) as a preprocessing step to reduce the target area; then, a combination of maximal similarity-based region merging (MSRM) and the fast-marching edge-based algorithm is introduced to improve the robustness and accuracy of segmentation edges. Wei et al. [33] proposed a threshold method using Otsu’s thresholding algorithm and a filtering process to achieve easy, fast, and effective segmentation results in tongue diagnosis. Fachrurrozi et al. [34] applied the harmony search algorithm (HSA) to optimize the obtained threshold and assist Otsu in optimizing the output results. Wei et al. [35] used quad-tree decomposition to divide collected tongue images. In this method, the mean pixel values of similar areas are used to represent the pixels in the area in order to estimate the Gaussian mixture model (GMM) parameters, with the GrabCut algorithm then used to segment the tongue image. While these tongue image segmentation algorithms based on traditional methods have achieved good results, there are still a number of limitations. They are sensitive to the quality of the dataset, and are easily affected by tongue image features such as light changes, tongue posture, tongue exposure, and other factors. These features lead to a decrease in the segmentation effect, requiring researchers to control the quality of feature extraction and set thresholds.

### 2.2. Deep Learning Segmentation

The development of deep learning methods has brought new possibilities to medical image processing. Wang et al. [36] used a fully convolutional network (FCN) to segment tongue images, achieving greater improvement in accuracy compared to traditional segmentation methods such as Otsu and GrabCut. Huang et al. [37] proposed the TISNet segmentation algorithm, using ResNet101 as the encoder for feature extraction and combining the receptive field blocks with multi-branch convolution blocks and shortcut links to extract contextual information. Feature fusion was performed using a feature pyramid as the decoder. After the UNet model began to be applied to image segmentation, several tongue image segmentation works based on UNet were proposed. Zhang et al. [38] introduced the ResNet18 residual structure as the encoder to extract features and obtain rough segmentation results based on the UNet structure. Next, superpixel image segmentation was performed and the segmentation edges were refined by updating the pixel categories. Under the multi-task learning framework, Xu et al. [39] used UNet for feature extraction and discriminative filter learning (DFL) for fine-grained classification. By sharing parameters, they simultaneously completed the tasks of tongue image segmentation and tongue image classification. Song et al. [40] optimized the encoder of the UNet network by fusing the squeeze-and-excitation (SE) blocks between the residual blocks of ResNet, enhancing the feature extraction capability of the encoder. Then, they completed the optimization by introducing a weighted cross-entropy loss function. Peng et al. [41] simplified the UNet structure and proposed P-Net, which deletes the last layer of the original decoder and instead sends the feature map of the input image to the beginning of the encoder and the end of the decoder. The dual attention gate module is used for information fusion to enhance boundary attention. Zhang et al. [16] adjusted the atrous convolution scale of the ASPP module based on DeeplabV3+, reducing the rate of the three-scale atrous convolution module in equal proportions and improving its ability to extract multi-scale information. To address the problem of unclear segmentation from the lips and teeth, a cross-entropy loss function that fuses edge information was proposed. Jiang et al. [17] used the Mask-RCNN framework to segment the tongue area and the Faster-RCNN framework to identify the tongue shape and texture in order to assist in the diagnosis of nonalcoholic fatty liver disease (NAFLD). While these tongue image segmentation methods based on deep learning have further improved the segmentation effect, the segmentation objects still focus on high-quality tongue images. For tongue images under complex conditions, there is still room for improvement in processing the interface between the tongue, lips, and teeth.

## 3. Methods

**Figure 1:**
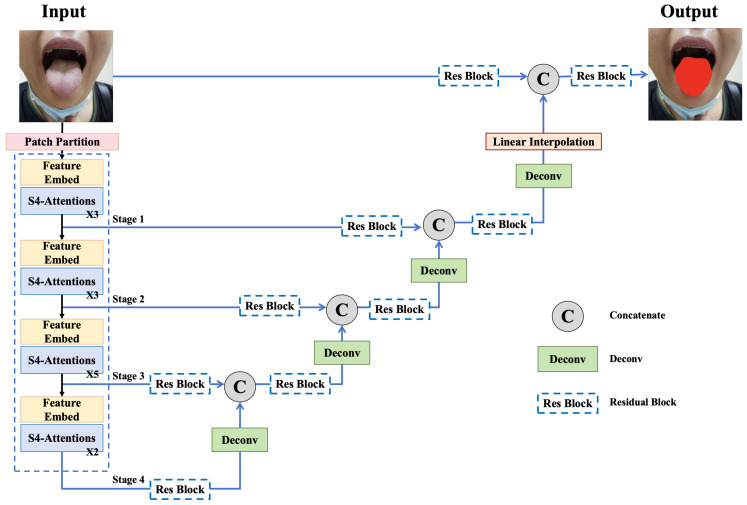
Overview of S5Utis architecture.

An excellent segmentation model needs to be based on an efficient backbone. The S5Utis tongue segmentation network is mainly divided into three parts: an encoder, a decoder, and S4-2D modeling for image data. The encoder introduces the structure from ViT [42] and SegNeXt [26] for multi-scale fusion. The attention block is improved by the S4-2D layer [43] for modeling the underlying signals of the image data. To capture the high-level semantics and ensure that segmentation details are not lost, a decoder with a skip connection and layer-by-layer upsampling is applied [44]. Meanwhile, a custom loss function is designed to optimize the network parameters by combining a common cross-entropy loss function and boundary loss function [45]. However, after testing, the modified loss function failed to provide better performance. Thus, in the formal version the S5Utis model still uses the traditional cross-entropy loss function. The network structure of S5Utis is shown in Figure 1.

### 3.1. Encoder

In current research, networks with good segmentation effects such as UNet [46] and DeeplabV3+ [47] are already in use and have undergone relevant improvements to make them better adapted to the task of tongue image segmentation. However, the feature extraction ability of the UNet network’s encoder is insufficient, and the network may not be able to handle targets of different sizes because the feature map size between the encoder and the decoder is equal. DeeplabV3+ can better capture feature information at different scales and improve segmentation effects due to the introduction of the ASPP module and dilated convolution; however, its introduction results in a surge in the number of calculations, and it has difficulty dealing with edge blur. SegNeXt is a simple convolutional network based on semantic segmentation that makes extensive use of depth-wise convolution, which can reduce the number of parameters and better encode contextual information to complete the semantic segmentation task. Considering that SegNeXt is more capable of handling multi-scale information and feature fusion compared to the currently popular DeeplabV3+ and UNet networks, we use SegNeXt as the encoder in the improved S5Utis model; the S4-ND block replaces the 1×1 convolution in the SegNeXt attention module, thereby enhancing the encoder’s ability to generalize across multiple resolutions. Here, ND stands for the number of dimensions.

**Figure 2:**
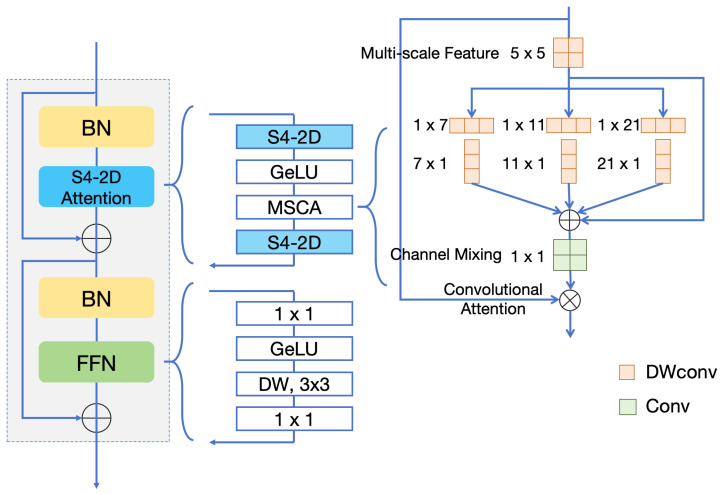
S4-Attention block of the S5Utis model’s modified encoder structure.

We consider that tongue images can be modeled as discretizations of inherently continuous multidimensional signals. In the following description, 2D is used to instead of ND to indicate that our modeling object is an image. S4-2D is a new multidimensional SSM layer that extends the continuous signal modeling capabilities of SSM to two-dimensional data such as tongue images. S4-2D implicitly learns global continuous convolutional kernels that are constructed to be resolution-invariant, thereby providing inductive bias. S4-2D is able to generalize across multiple resolutions. The modified structure of the encoder is shown in Figure 2. The whole encoder consists of four stages. The number of building blocks in each channel is 3, 3, 5, and 2, respectively, as shown in Table 1.

### 3.2. S4-2D Block

#### 3.2.1. State Space Models

State space models (SSMs) are widely used in many fields. Such models describe a system by determining its state variables. They can be applied to both dynamic systems and complex systems. When applied to the field of deep learning, they can help to capture long-term dependency problems in data. The formulas is defined below.

(1) $$\begin{array}{c} x^{\prime }\left(t\right)=Ax\left(t\right)+Bu\left(t\right) \end{array}$$

(2) $$\begin{array}{c} y(t)=Cx(t)+Du(t) \end{array}$$

**Figure 3:**
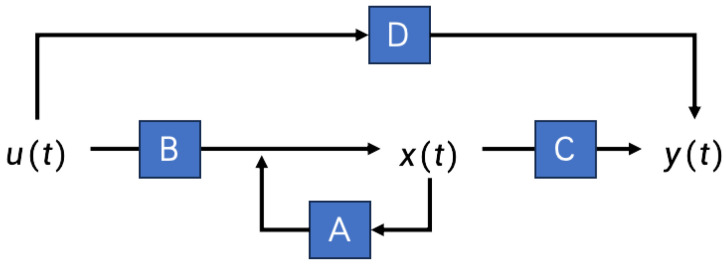
SSM structure.

An SSM is divided into a state Equation (1) and observation Equation (2). The state equation describes the changes in the system state over time, while the observation equation describes the relationship between the system’s output and its state. This relationship reflects the status of the system. In an SSM, u(t) is the input sequence, y(t) is the output sequence, and x(t) is the potential state of the input sequence mapped to *N* dimensions, that is, the system state; $x^{\prime }\left(t\right)$ is the derivative of state x(t) with respect to time *t*, *A* is the state transition matrix, *B* is the input matrix, *C* is the output matrix (observation matrix), and *D* is the feedforward matrix (generally, *D* takes a value of 0). A diagram of the SSM structure is shown in Figure 3.

#### 3.2.2. HIPPO Matrix and S4 Structure

Based on SSM, Albert Gu and others proposed the HiPPO (High-order Polynomial Projection Operators) architecture [48]. To solve the long-term dependency problem, HiPPO compresses historical records and generates the optimal solution of the state matrix *A* in the SSM through function approximation. In order to better apply the above SSM to deep learning, HiPPO uses the zero-order hold (ZOH) method to discretize the SSM. This discretization can also reduce the complexity of the model. The resulting discretized SSM can be expressed as follows:

(3) $$\begin{array}{ccc} \; & x_{k}=\bar{A}x_{k-1}+\bar{B}u_{k} & \\ \; & y_{k}=\bar{C}x_{k} & \\ \; & \bar{A}={\left(I-\Delta /2\cdot A\right)}^{-1}\left(I+\Delta /2\cdot A\right) & \\ \; & \bar{B}={\left(I-\Delta /2\cdot A\right)}^{-1}B & \\ \; & \bar{C}=C \end{array}$$

where Δt is the discrete time step. At each time step, the SSM calculates how the current input $\bar{B}u_{k}$ affects the previous state $\bar{A}x_{k-1}$, then predicts the output $\bar{C}x_{k}$ through the discretized state equation.

The HiPPO architecture compresses all input signals before time *t* into coefficient vectors, then uses the state matrix *A* to store the latest token, attenuates the old token, and generates the optimal solution of the state matrix *A* through function approximation. At this time, the state matrix *A* becomes the HiPPO matrix. Matrix *A* and matrix *B* can be expressed as follows.

(4) $$A_{nk}=\left\{\begin{array}{cc} {\left(2n+1\right)}^{1/2}{\left(2k+1\right)}^{1/2} & if\;n>k\\ n+1 & if\;n=k,\;B_{n}={\left(2n+1\right)}^{1/2}\\ 0 & if\;n<k \end{array}\right.$$

The HiPPO matrix completes the approximation of all past historical records by tracking the coefficients of the Legendre polynomial. Therefore, in practical applications, long-term dependency problems can be handled well even in cyclic structures similar to RNN. In order to preserve sequence history information, orthogonal polynomials are used to project historical data in HiPPO and converted into SSM form with special initialization matrices *A* and *B*.

After discretization, the SSM is similar to an RNN; however, compared with RNNs, the discretized SSM can better compress and memorize historical states. According to Equations (1) and (2), the linear ODE can be converted to a convolutional form. Equation (3) can be written as a discrete convolution. When the initial state is set as $x_{-1}=0$, the unrolling equation in Equation (3) can be written as follows.

$$\begin{array}{cc} \; & x_{0}=\bar{B}u_{0}\;x_{1}=\bar{AB}u_{0}+\bar{B}u_{1}\;x_{2}={\bar{A}}^{2}\bar{B}u_{0}+\bar{AB}u_{1}+\bar{B}u_{2}\;\cdots \\ \; & y_{0}=\bar{CB}u_{0}\;Y_{1}=\bar{CAB}u_{0}+\bar{CB}u_{1}\;y_{2}=\bar{C}{\bar{A}}^{2}\bar{B}u_{0}+\bar{CAB}u_{1}+\bar{CB}u_{2}\;\cdots \end{array}$$

Then, it is possible to represent $y_{k}$ as follows:

(5) $$\begin{array}{ccc} \; & y_{k}=\bar{C}{\bar{A}}^{k}\bar{B}u_{0}+\bar{C}{\bar{A}}^{k-1}\bar{B}u_{1}+\cdots +\bar{CAB}u_{k-1}+\bar{CB}u_{k} & \\ \; & y=\bar{K}*u & \end{array}$$

where $\bar{K}\in R^{L}$ is the kernel of the convolution, which is a linear combination of basis kernels ${\bar{K}}_{n}={\bar{A}}^{i}\bar{B},i\in \left[L\right]$:

(6) $$\begin{array}{cc} \bar{K}\in R^{L}:=K_{L}\left(\bar{A},\bar{B},\bar{C}\right) & =(\bar{CB},\bar{CAB},\cdots ,\bar{C}{\bar{A}}^{L-1}\bar{B})\\ \; & =\bar{C}\left(\bar{B},\bar{AB},\cdots ,{\bar{A}}^{L-1}\bar{B}\right)\\ \; & =\bar{C}\left({\bar{K}}_{0},{\bar{K}}_{1},\cdots ,{\bar{K}}_{i}\right). \end{array}$$

**Figure 4:**
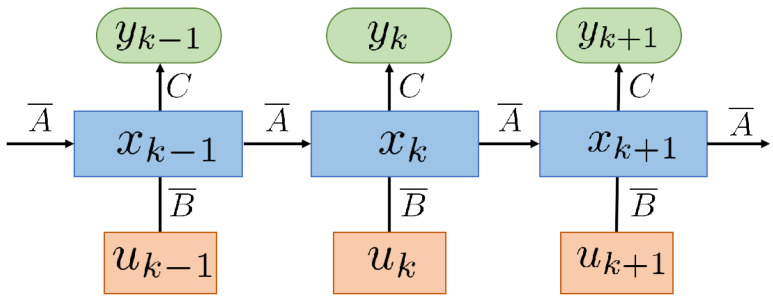
Unfolded SSM.

The specific structure is shown in Figure 4.

In the S4 architecture, the HiPPO matrix *A* has been further optimized with the aim of diagonalizing the matrix *A*. After diagonalization, the computational complexity of SSM changes from $O(N^{2})$ to O(N); however, direct diagonalization leads to numerical overflow. Therefore, the diagonalization of *A* is completed by combining a regular matrix with a low-rank matrix. The transformed representation of the HiPPO matrix *A* is

(7) $$A=V^{-1}\Lambda V-PQ^{T}=V^{-1}\left(\Lambda -\left(VP\right)\left(P^{T}V^{-1}\right)\right)V,$$

where Λ is a diagonal matrix, $V\in R^{N\times N}$, and $P,Q\in R^{N\times r}$ is a low-rank matrix. After decomposition, the computational complexity of S4 is reduced. The recursive calculation complexity of S4 is O(N)MVM, where MVM represents matrix vector multiplication. The convolution complexity is reduced from $O(LN^{2})$ to O(L+N) via Cauchy matrix vector multiplication, with *N* representing the hidden dimension and *L* the sequence length.

#### 3.2.3. S4 for 2D Input Image

**Figure 5:**
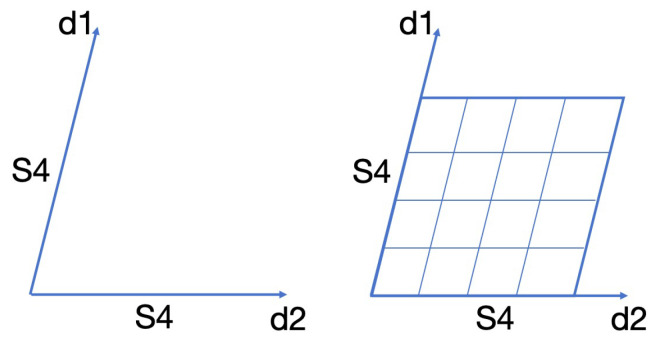
2D-S4 is used to model the images as 2D inputs.

We generalize the SSM in Equations (5) and (6) to two dimensions [43]. Here, we denote the two spatial dimensions of the 2D input image as $d_{1}$ and $d_{2}$, respectively. The 2D convolution $y=\bar{K}*u$ can be represented as follows:

(8) $$\begin{array}{c} {\bar{K}}^{\left(d_{1},d_{2}\right)}=\langle \bar{C},\left({\bar{K}}_{i}^{\left(d_{1}\right)}{\bar{B}}^{\left(d_{1}\right)}\right)⊗\left({\bar{K}}_{i}^{\left(d_{2}\right)}{\bar{B}}^{\left(d_{2}\right)}\right)\rangle \end{array}$$

where ${\bar{K}}_{i}^{\tau }$ are the standard 1D SSM kernels for each axis. As shown in Figure 5, two independent S4 kernels are instantiated to represent the entire input lengths of each dimension and produce a global convolutional kernel by computing the outer product.

### 3.3. Decoder

Using SegNeXt as the backbone of the segmentation network can effectively improve the accuracy and efficiency of tongue image segmentation. However, the Hamburger module used in the decoding part of SegNeXt loses the features in the initial stage during the decoding process, which is not conducive to retaining more segmentation details. Therefore, we considered using cross-layer connections and progressive upsampling to ensure that the details of the original image are not lost during the transmission process.

We redesigned the decoder part suitable for the tongue image segmentation task based on UNet and UNETR, then connected the encoder to the decoder through skip connections. A residual block was introduced to directly connect the output of each stage with the feature map of the previous layer to improve the feature fusion. The modified structure of the decoder is shown in the overview of the S5Utis architecture in Figure 2.

### 3.4. Loss Function

Cross-entropy loss functions are commonly used in image segmentation models. Considering the segmentation task’s focus on segmentation boundaries, we explored introducing a boundary-based loss function. The boundary loss function was proposed by Kervadec et al. [45]. In our experiment, we used the cross-entropy loss function in conjunction with the boundary loss function. The boundary loss function was designed as follows:

(9) $$\begin{array}{c} L_{B}\left(\theta \right)=\int_{\Omega }\phi_{G}\left(q\right)s_{\theta }\left(q\right)dq \end{array}$$

where Ω is the spatial domain of the train image, *G* is the ground truth region, *S* is the predicted region, ∂G is the ground truth boundary, and ∂S is the predicted boundary. Here, q∈Ω is a point in ΔS, with ΔS being the region between *G* and *S* and with $D_{G}\left(q\right)$ representing the distance between any point *q* and the nearest point $z_{\partial G}\left(q\right)$ on contour ∂G.

## 4. Results and Discussion

### 4.1. Dateset and Implementation Details

The tongue image segmentation dataset was taken from the Guangdong Provincial Hospital of Traditional Chinese Medicine and the First Affiliated Hospital of Guangzhou University of Chinese Medicine. The ethics committee reviewed the experiment and data collection methods. The tongue image dataset consisted of 416 images; all tongue imaging data were anonymized. The tongue image data were collected between 2021 and 2022, and consisted of tongue images taken by doctors or patients using mobile phones. There were no standardized light sources or shooting angle requirements. All tongue images were taken in real environments. The tongue image data segmentation label results were proofread and approved by doctors specializing in TCM.

The dataset was divided into training and test sets using a ratio of 8:2. No data augmentation approach was used during training. The tongue images taken by non-professionals were directly used for training and testing.

The models were implemented using PyTorch 1.13.1 and trained on one NVIDIA GeForce RTX 3090 GPU. All models, including baseline models, were trained from scratch on the same TCM dataset without pretraining. The optimizer used by the proposed S5Utis model is AdamW. The hyperparameters used in the training process of the S5Utis model can be found in Table 2.

### 4.2. Experimental Results and Analysis

To evaluate the effectiveness of the S5NeXt model, UNet [46], SETR [49], Segformer [50], UNETR [44], and SegNeXt [26], all of which are widely used in daily image segmentation and medical image segmentation tasks, were selected as comparison models.

In the proposed model’s quantitative evaluation process, three commonly used evaluation metrics in image segmentation were selected to evaluate the effect of tongue image segmentation: the Dice similarity coefficient (F1-Score), mIoU coefficient (mean intersection over union), and pixel accuracy (PA). True positives (TP) are pixels that are considered to be inside the target region and are actually inside the target region. True negatives (TN) are pixels that are considered to be outside the target region after segmentation and are actually outside the target region. False positives (FP) are pixels that are considered to be inside the target region after segmentation but are actually not. False negatives (FN) are pixels that are considered to be outside the target region after segmentation but are actually inside the target region.

(10) $$\mathrm{Dice}=\frac{2\times \mathrm{TP}}{\mathrm{FP}+2\times \mathrm{TP}+\mathrm{FN}}$$

(11) $$\mathrm{mIoU}=\frac{1}{2}\left(\frac{\mathrm{TP}}{\mathrm{TP}+\mathrm{FP}+\mathrm{FN}}+\frac{\mathrm{TN}}{\mathrm{TN}+\mathrm{FN}+\mathrm{FP}}\right)$$

(12) $$\mathrm{PA}=\frac{\left(\mathrm{TP}+\mathrm{TN}\right)}{\left(\mathrm{TP}+\mathrm{TN}+\mathrm{FP}+\mathrm{FN}\right)}$$

The proposed S5Utis model was compared with the popular segmentation algorithms listed above. The cross-entropy loss function, abbreviated as CE, was used for all the models, including the proposed S5Utis model. The evaluation of the segmentation results is shown in Table 3.

The experimental results show that our proposed method achieves the best results compared to baseline methods. In the internal comparison of baseline methods, based on the results of Segformer and UNTRE, the transformer structure seems to perform poorly in the three evaluation indicators. The performance of traditional SegNeXt is better, which shows that the SegNeXt framework has the potential to improve tongue image segmentation. After improving SegNeXt by adding the S4-2D layer and optimizing the decoder, our S5Utis algorithm achieves the best results among all comparison indicators, indicating that our algorithm is capable of segmenting tongue images in real environments.

### 4.3. Ablation Experiment Results

We conducted ablation experiments on the improved elements of the model in order to verify the effectiveness of the improvements, with the results shown in Table 4. First, we conducted an ablation experiment on the S4-2D module. It was clearly observed that the improved UNETR achieved a better result after adding the S4-2D module. However, the improved SegNeXt with the S4-2D module did not achieve positive results on all three evaluation indicators; while the Dice and PA results were better than the original model, the mIoU was not as positive as expected. The positive impact of the S4-2D module is mainly reflected in accuracy, which is consistent with previous research results. This shows that although the addition of the S4-2D module has an effect on improving the segmentation effect, this positive effect may not be fully reflected when used independently. Thus, other structures such as decoders need to be simultaneously improved in order to achieve better results.

Subsequently, we compared the results with the encoder module, finding that the image segmentation quality improved with the updated decoder. From the results in Table 4, it can be seen that after updating the encoder, all three evaluation indicators improve, achieving the best results among all current methods.

We additionally conducted ablation experiments using the modified loss function. However, the combination of the cross-entropy function (CE) and boundary function (BL) did not have a positive effect on the segmentation. As can be seen from Table 5, adding the boundary function has no positive effect on the three evaluation indicators. Therefore, in the formal S5Utis model we used the cross-entropy loss function instead of the modified loss function, as shown in Table 3 and Table 4.

### 4.4. Tongue Segmentation Visualization

In order to display the segmentation results more intuitively, we selected several representative tongue images to compare the segmentation effects of different methods. These tongue images included instances of twisted tongue, tooth interference, abnormal tongue coating color, pigment spots on the tongue, and tooth-marked tongue.

**Figure 6:**
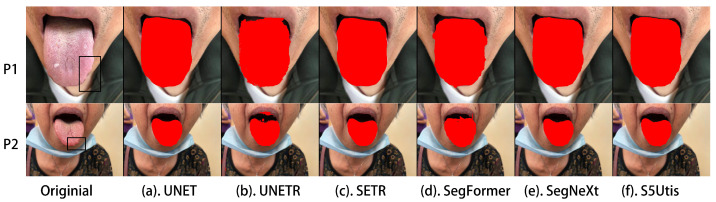
Tooth-marked tongue: segmentation results of different methods.

**Figure 7:**
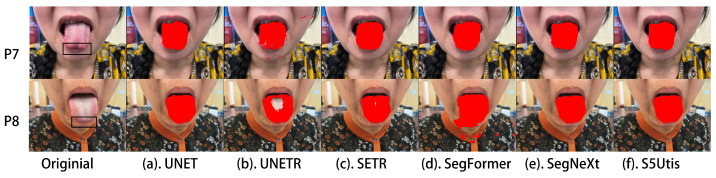
Inward curvature at the tip of the tongue: segmentation results of different methods.

First, we visualized the abnormal tongue shapes. Figure 6 shows an instance of tooth-marked tongue in the TCM tongue shape classification, which is characterized by an unsmooth edge that resembles a tooth mark. Figure 7 shows a tongue with an inward curvature at the tip of the tongue, similar to an unfolded letter ’w’. From the visualization results, it can be seen that the proposed method is able to identify these unsmooth and irregular edges, helping to obtain tongue segmentation results that are closer to reality. At the same time, the tongue coating of patient P8 is white, which has a certain impact on the segmentation of tongue images. For example, the UNETR algorithm cannot recognize the tongue coating at all. However, the proposed method can identify both the tongue coating with abnormal color and segment the shape of the tongue tip.

**Figure 8:**
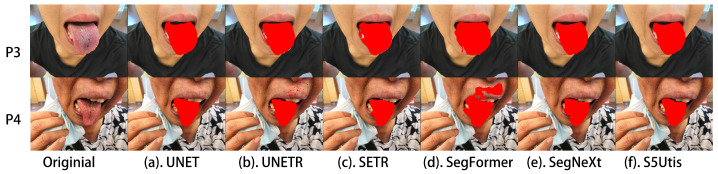
Twisted tongue: segmentation results of different methods.

As it may be the case that neither the photographer nor the patient are professional medical personnel, the tongue may be twisted when taking the tongue image, resulting in insufficient exposure of the tongue. The resulting images are different from normal tongue images, as shown in Figure 8. Our algorithm is able to identify and segment these tongue images with twisted shapes very well.

**Figure 9:**
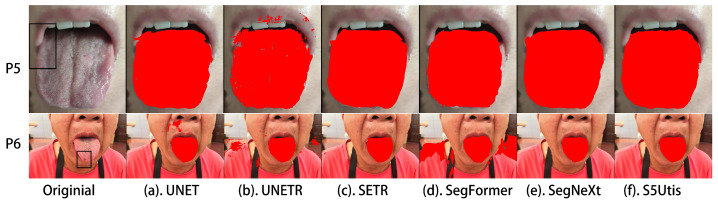
Noise interference of lips and pigment spots: segmentation results of different methods.

In addition, the color of the lips may be similar to that of the tongue, which often affects the segmentation effect in segmentation tasks. In addition to tongue coatings of different colors, there are occasionally abnormal color areas such as pigment spots on the tongue, exemplified by patient P6 in Figure 9. Compared with other baseline methods, our proposed algorithm can better handle the impact of lip color, as in the example of patient P5, and can identify pigment spots on the tongue as normal tongue tissue, such as for patient P6.

By comparing these results, it can be seen that our proposed method adequately addresses the problems present in low-quality tongue images taken by untrained patients. It is barely affected by complex background factors such as environment, clothes, face, lips, etc., and correctly segments the images even in the presence of such noise.

**Figure 10:**
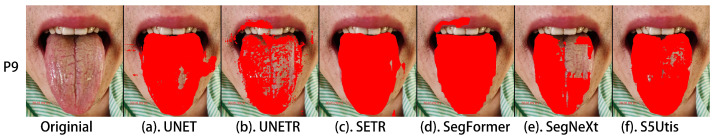
Failure case: segmentation results of different methods.

However, there is room for improvement in the tongue image segmentation algorithm. In our experiments, we found segmentation failure samples, as shown in Figure 10. Through the visualization results, we found that the SETR and SegFormer algorithms are superior to other baseline methods, including our proposed method, in terms of tongue surface integrity. We consider that this may be due to the decoder part of SETR and SegFormer not using skip connections, instead performing layer-by-layer merging or upsampling after the output. Other poorly performing algorithms use skip connections between the encoder and decoder, which can retain high-resolution features in the deep layer of the network. Thus, we consider that the feature fusion after the jump connection is over-learned, resulting in the failure of pixel classification. In future work, it will be necessary to further explore the decoder part and improve the segmentation effect by optimizing the structure of the jump connection.

## 5. Conclusions

This paper proposes a tongue image segmentation method called S5Utis for use in Traditional Chinese Medicine. The proposed method is based on the S4-2D module and an improved UNETR decoder under the SegNeXt framework. Experiments were conducted on a tongue image dataset jointly collected by doctors and patients using non-professional equipment in real environments, and the results were compared with existing semantic segmentation algorithms. The results show that our proposed method performs better in terms of various evaluation indicators and actual segmentation effects. Our proposed method is adaptable to image data collected in non-professional environments with non-professional equipment, and can play a positive role in promoting smart TCM in the future. By helping people to obtain better quality TCM diagnosis and recommendations in their daily health care activities, the proposed method is beneficial to maintaining long-term health.

## Figures and Tables

**Table 1:** Detailed settings of S5Utis. In the table, e.r. represents the expansion ratio in the feed-forward network, *C* is the number of channels, and *L* is the number of building blocks.

Stage	Output Size	e.r.	S5Utis
1	$\frac{H}{4}\times \frac{W}{4}\times C$	8	C=32,L=3
2	$\frac{H}{8}\times \frac{W}{8}\times C$	8	C=64,L=3
3	$\frac{H}{16}\times \frac{W}{16}\times C$	4	C=160,L=5
4	$\frac{H}{32}\times \frac{W}{32}\times C$	4	C=256,L=2

**Table 2:** Hyperparameter configuration used for the experiments.

Hyperparameter	Parameter Setting
Base_lr	0.0001
Beta_1	0.9
Beta_2	0.999
Momentum_1	0.9
Momentum_2	0.999
Batch_size	16
Droppath_rate in Encoder	0.01
BN_epsilon in Encoder	0.00001
BN_momentum in Encoder	0.1

**Table 3:** Results of experimental comparison of tongue image segmentation methods.

Model	Loss	Dice (%)	mIoU (%)	PA (%)
UNet	CE	96.08	93.37	98.37
UNet(S4ver)	CE	97.20	94.88	98.91
UNETR	CE	90.98	85.14	95.71
SETR	CE	94.76	91.01	97.61
Segformer	CE	87.39	80.51	93.76
SegNeXt	CE	97.20	94.80	98.81
**S5Utis**	CE	**98.01**	**96.18**	**99.21**

**Table 4:** S5Utis ablation experiment results (SegNeXt(S4ver) + UNETR is the full version of S5Utis).

Encoder	Decoder	Dice (%)	mIoU (%)	PA (%)
UNet	NONE	96.08	93.37	98.37
UNet(S4ver)	NONE	97.20	94.88	98.91
SegNeXt	HAM	97.20	94.80	98.81
SegNeXt(S4ver)	HAM	97.22	94.50	98.82
SegNeXt	UNETR	97.98	96.15	99.21
**SegNeXt(S4ver)**	**UNETR**	**98.01**	**96.18**	**99.21**

**Table 5:** Loss function ablation experiment results.

Model	Loss	Dice (%)	mIoU (%)	PA (%)
S5Utis	CE	**98.01**	**96.18**	**99.21**
S5Utis	CE + BL	97.78	95.77	99.14

## Data Availability

The data presented in this study are available on request from the corresponding author. The data are not publicly available due to privacy concerns.
